# Influence of Cross-Section Shape and FRP Reinforcement Layout on Shear Capacity of Strengthened RC Beams

**DOI:** 10.3390/ma15134545

**Published:** 2022-06-28

**Authors:** Muhammad Ahmed, Piero Colajanni, Salvatore Pagnotta

**Affiliations:** Dipartimento di Ingegneria, Università di Palermo, 90128 Palermo, Italy; muhammad.ahmed@unipa.it (M.A.); piero.colajanni@unipa.it (P.C.)

**Keywords:** FRP, shear strengthening, average shear strength, effectiveness factor, inclination

## Abstract

The evaluation of the shear capacity of an FRP-strengthened reinforced-concrete beam is challenging due to the complex interaction between different contributions provided by the concrete, steel stirrup and FRP reinforcement. The shape of the beam and the FRP inclination can have paramount importance that is not often recognized by the models that are suggested by codes. The interaction among different resisting mechanisms has a significant effect on the shear capacity of beams, since it can cause a reduction in the efficiency of some resisting mechanisms. A comparative study of the performance in the shear resistance assessment provided by three models with six different effectiveness factors (*R*) is performed, considering different cross-section shapes, FRP wrapping schemes, inclination and anchorage systems. The results revealed that the cross-section shape, the FRP inclination and the efficiency of the FRP anchorages have a significant effect on the shear strength of beams. The analysis results show that the three models are able to provide an accurate average estimation of shear strength (but with a coefficient of variation up to 0.35) when FRP reinforcement orthogonal to the beam axis is considered, while a significant underestimation (up to 19%) affected the results for inclined FRP reinforcement. Moreover, all the models underestimated the resistance of beams with a T section.

## 1. Introduction

Shear failure in RC members is one of the most critical and undesired failure phenomena. Beams and columns of existing RC Moment Resisting Frames (MRFs) usually do not satisfy the current code requirements regarding shear strength; thus, often it becomes necessary to strengthen the existing RC structural member in order to protect it from unwanted shear failure [1,2].

Over the last two decades, innovative strengthening techniques such as the use of externally bonded (EB) or near-surface-mounted (NSM) fiber-reinforced polymer (FRP) and externally bonded fiber-reinforced cementitious matrix (FRCM) have been widely used in the axial and shear strengthening of RC members [3,4].

Yet, it is quite difficult to accurately design a strengthening intervention of RC members by means of externally bonded FRP because member strength evaluation is still a topic of debate [5,6]. Many experiments confirm that the shear failure of FRP-strengthened beams usually occurs due to debonding of the FRP [7,8], but different failure modes can occur in a strengthened RC member due to the very presence of the FRP, which is affected by brittle failure [9].

The shear strength evaluation of FRP-reinforced RC beams is also quite complex due to the presence and interaction of the three main contributors, i.e., concrete, externally bonded FRP and transverse steel reinforcement. Experimental results have proved that the presence of FRP modifies the shear contributions provided by concrete and transverse steel reinforcement [10]. 

In this connection, the brittle failure of FRP reinforcement, which can occur before the yielding of steel stirrups, can have a negative effect on the shear strength provided by the transverse steel reinforcement [11,12]. Some researchers have proved that, when members having a significant amount of steel stirrups have to be strengthened, the transverse reinforcement provides a greater contribution than the FRP because the bond between the steel and concrete is stronger than between the FRP and the concrete surface [13]. However, experiments have also revealed that sometimes the efficiency of transverse reinforcement decreases with the presence of FRP due to brittle failure of the latter, which hinders the yielding of all the steel stirrups intersected by the shear-critical crack, as well as limits the strain achieved by the stirrups at failure [12,13].

The shear contribution provided by the FRP depends on the strengthening scheme. It can be done in different ways: (1) complete wrapping (C) of the member; (2) partial wrapping (U-shape); (3) side wrapping [14]. U-shape and side wrapping are more prone to debonding failure, while there are negligible chances of debonding failure for completely shaped wrapping. The side-wrapping scheme is not considered here because it does not provide a significant increment in the shear capacity of FRP-strengthened RC beams. To avoid debonding failure, a proper anchorage length or proper mechanical connector that prevents debonding between the FRP and the concrete surface should be provided [15].

In the case of the U-shaped scheme, several researchers have introduced different types of anchorages for FRP which have proved to be effective in increasing the shear contribution of the FRP [12,16]. However, their effectiveness is significantly variable due to different arrangements and other technological issues.

Two different approaches are pursued by codes for the shear resistance evaluation of strengthened members. According to a first group of international codes (CSA 2006 [17], ACI 440.2R/17 [18]), the strength of the RC beam reinforced by FRP is evaluated by an additive method. The overall shear resistance of the RC beam is considered as the sum of *V_c_* (shear resistance of concrete), *V_s_* (shear resistance of steel stirrups) and *V_f_* (shear resistance provided by FRP). Regarding the last two contributions, each of them is evaluated by separately taking into account the orientation of each reinforcement, namely (*β*) for the FRP and (*α*) for the transverse reinforcement.

By contrast, according to the European approach, the contributions of all the components are considered using the truss mechanism with variable inclination of the concrete strut. Thus, the inclination angles of the FRP (*β*) and transverse reinforcement (*α*) are parameters of paramount importance, since they affect the shear strength and contribution of all three of the components discussed above.

Colajanni et al. [19] analyzed a large database comparing the experimental shear and the analytically calculated shear strength of different models. It was found that the angle of inclination of the FRP has a significant effect on the shear strength of RC beams. Moreover, by changing the inclination angle of the FRP with respect to the beam axis, there is a significant change in the interaction between the *V_f_* and *V_s_*. 

Oller et al. [20] found that there is some difference between the sum of *V_f_*, *V_s_*, and *V_c_* and the total shear force. The experimental results show that there is a significant contribution of the flange to the shear strength in the case of T-cross-sectional members. In some cases, it was found to be up to 45% of the total shear strength. None of the code models recognizes the effect of the flange in a T cross section as relevant in modifying member shear resistance.

However, despite the presence of such a complex framework, it is unanimously recognized that among the factors affecting the estimation of shear strength, the effective strain of the FRP, expressed through the reduction factor *R*, plays a predominant role. 

In the light of the foregoing discussion, three main models for an RC shear critical beam strengthened with FRP are considered in this research: the model of Colajanni et al. [19], ACI 440.2R-17 [18] and CNR-DT-200/R1 [21]. Each model is analyzed with six different formulations that incorporate the effective FRP strain (*R* factor). Two of the approaches, namely (1: Khalifa and Nanni (2000, 2002), Pellegrino Modena (2006), and 2: Chen and Teng (2003)) [7,22,23,24,25] are adopted in the Colajanni et al. model, which also considers the reduction factor for steel stirrups [19].

These models are well suited for rectangular RC beams, but the effectiveness in calculating the shear strength of T sections is still a matter of discussion, since the effect of the contribution of flanges is not incorporated in all of them.

To fully understand the effect of a cross-section shape and the influence of the inclination angle of FRP on the shear resistance of the strengthened beam, experimental results on strengthened beam specimens with different section shapes or FRP inclination angles and equal values of the other geometrical and mechanical parameters should be available. Failing these, the influence can be indirectly detected by the variation in the ability of the different analytical models to predict the experimental results. 

To this aim, a large database was collected, considering rectangular and T sections, with vertical and inclined FRP, also including specimens characterized by mechanical anchorages between FRP and the RC beam. The experimental values were compared with the shear-strength values obtained from the three above-mentioned models [18,19,21]. The results are discussed focusing on the influence of the cross-section shape and the FRP angle of inclination on both the shear strength of the specimen and the reliability of the shear models, also considering the effect of the various effectiveness-factor models for FRP.

Two different analyzing approaches were adopted. The first approach was to cover the influence of the cross-section shape on the shear strength of the beam externally bonded by FRP. Then a comparison was made between the assessment of the shear strength of R and the T-cross-sectional members with the FRP and steel web reinforcement having the same inclination (*α = β*) by means of a fixed model. To cover the influence of FRP inclination, a comparison was made between the same shape but with different inclinations (*α* ≠ *β*), again by means of a fixed model. 

## 2. Shear Models

Three different models—ACI 440.2R-17 [18], CNR-DT-200/R1 [21] and Colajanni et al. [19]—are reported and discussed below. The code models are reported hereinafter as presented in their original form. It has to be stressed that in the shear prediction discussed in Section 6, neither the safety factors for the FRP reinforcement, steel stirrups and concrete were considered, nor the strength-reduction factor *φ*.

### 2.1. ACI 440.2R-17 

ACI 440.2R-17 [18] gives guidelines for the design and evaluation of the shear strength for an RC beam strengthened with externally bonded FRP based on an additive approach.

**Figure 1:**
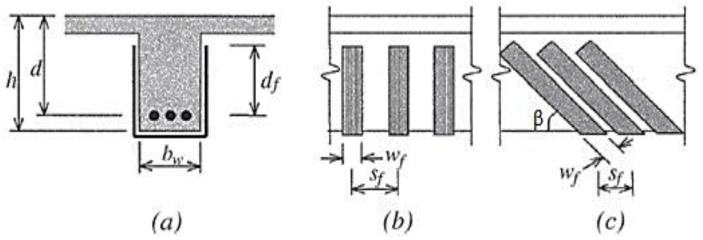
Variables used in the ACI 440.2R model for shear-strengthening calculations (**a**) cross-sectional parameters (**b**) spacing and width of FRP (**c**) inclination angle between FRP strip and beam axis.

ACI 318-14 (ACI 2014) [26] is used for the evaluation of concrete (*V_c_*)*,* steel stirrup (*V_s_*) and FRP (*V_f_*) contributions. According to the symbol notation in Figure 1 and Abbreviations, *V_c_* (nominal shear strength provided by shear reinforcement) is calculated as:

*V_c_* = 0.167 *f*′*c*^0.5^ *b_w_ d* (1)

while *V_s_* (nominal shear strength provided by shear reinforcement) is calculated as:

*V_s_* = (*A_v_ f_yt_ d*)/*s* (2)

and the shear strength provided by the FRP is calculated as:

*V_f_ =* (*A_fv_ d_fv_ f_fe_* (sin *β +* cos *β*))*/s_f_* (3)

In the *V_f_* equation, *A_fv_* = 2*nt_f_w_f_*, *f_fe_* = *ε_fe_ E_f_* and different safety factors are used for different wrapping schemes. The shear strength of the retrofitted RC beam is equal to:

*φV* = *φ* (*V_c_* + *V_s_* + *ψ_f_ V_f_*) (4)

For the completely wrapped scheme, *ψ_f_* = 0.95 while for other schemes *ψ_f_* = 0.85, while *φ* is a strength-reduction factor. The effective strain of the FRP (*ɛ_fe_*) is calculated based on the different configurations. It should not be more than 0.75 of the ultimate strain *ε_fu_,* while for the design it should be limited to 4 × 10^−3^. The FRP effective depth is considered as the distance between the centroid of tensile reinforcement and the top free edge of the FRP. It must be stressed that the ACI model takes into account the actual height of the FRP reinforcement by the parameter *d_fv_* (Figure 1). 

To obtain the effective strain *ε_fe_* of partly wrapped sections, the ultimate strain of the FRP is multiplied by a bond-reduction factor *k_v,_* as *ε_fe_ = k_v_ ɛ_fu_* ≤ 4 × 10^−3^. *k_v_* can be calculated as *k_v_ = k*_1_*k*_2_*L_e_*/(11,900*ɛ_fu_*) ≤ 0.75, where the modification factors *k*_1_ and *k*_2_ can be calculated by using *k*_1_ = (*f′_c_*/27)^2/3^ and *k*_2_ = (*d_fv_ − γL_e_*)/*d_fv_* (γ = 1 for the U-wrapped scheme and γ = 2 when both sides are wrapped), where the effective length is *L_e_* = 23,300/ (*E_f_ t_f_*)^0.58^.

### 2.2. CNR Model

The CNR-DT 200 R1/2013 [21] is the model established by the Italian Research Council (CNR) and it deals with two types of wrapping: U-shaped and full. The equations given are the extension of the equations provided in EN1992-1-1 [27] to evaluate the shear strength of reinforced-concrete beams. The model is derived according to the truss mechanism with variable inclination of the concrete strut, in which the shear capacity of the FRP is calculated using:

*V_Rd,f_* = (1/*γ_Rd_*) 0.9 *d f_fed_* 2 *t*_f_ (cot *θ +* cot *β*) (*b*_f_/*p*_f_)sin^2^ *β* (5)

Equation (5) is reported and used consistently with the equation reported in the new version of the CNR-DT code for strengthening by fiber/fabric-reinforced cementitious matrix/mortar [28].

In this equation, p*_f_* = $\bar{p}$*_f_* sin *β* represents the spacing of the FRP measured perpendicular to the direction of fiber. The shear capacity of the stirrup and concrete strut is given as:

*V_Rd,s_* = 0.9 *d* (*A_sw_*/*s*) *f_ywd_* (cot *θ* + cot *α*) sin *α* (6)

*V_Rd,c_* = 0.9 *d b α_c_* 0.5 *f_cd_* (cot *α* + cot *θ*)/(1 + cot^2^ *θ*) (7)

In Equation (7), *α_c_ =* 1 has to be retained for the beam, and the angle *ψ*, yet to be determined, can be introduced by replacing the angle *α* listed in the code, in order to stress that the evaluation of the shear strength of the compressed concrete strut is not a trivial issue, as will be shown below. The strengthened member shear resistance is computed as:

*V_Rd_* = min (*V_Rd,s_* + *V_Rd,f,_ V_Rd,c_*) (8)

In evaluating the shear strength in a beam with FRP reinforcement inclined with an angle *β* ≠ *α*, the CNR-DT 200 R1/2013 model assumes *ψ* = *β*, taking into account in the truss scheme the inclination of the FRP reinforcement (*β*) only. Thus, in evaluating the strength of the compressed concrete strut, the model neglects the presence of two orders of web reinforcements and the amount of their contributions.

In this regard in [19], it is shown that in a beam which is to be shear-strengthened in which the existing shear reinforcement provides a significant contribution to the shear strength as the FRP, the angle of inclination of the concrete strut should be evaluated assuming *ψ* as a weighted value between *α* and *β*, where *V_Rd,s_* and *V_Rd,f_* are the weights.

### 2.3. Colajanni et al. Model

Colajanni et al. [19] proposed a model with variable inclination of the compressed concrete action based on stress field theory. It was derived on the basis of a previous model for a concrete beam reinforced by stirrups with two different inclinations [29]. In the same paper, it was validated against experimental results on ordinary RC beams and hybrid steel-trussed concrete beams (HSTCBs) [30].

**Figure 2:**
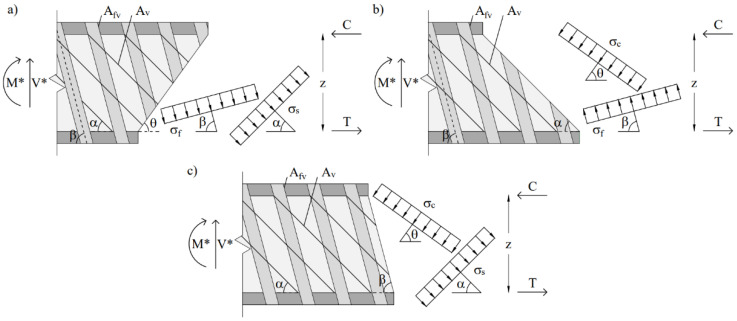
Beam segments identified via three sections parallel to stress field directions of (**a**) concrete strut; (**b**) steel stirrups; (**c**) FRP reinforcement. M* and V* represent the moment and shear acting on the considered section.

This model is able to correctly represent the shear strength of FRP-strengthened RC beams with shear reinforcement arranged in any direction. In order to evaluate the shear strength of the member, three different segments of beams are selected based on the stress field direction. They are obtained by sections parallel to the stress field of the FRP, concrete strut and steel stirrups which are demonstrated in Figure 2. The shear strength is calculated using three different equations by evaluating the vertical equilibrium of each beam segment:

(9) $$V\;=\;\left(b\;0.9\;d\;0.5\;f_{c}\right)\;\left\{\left[R{\tilde{\sigma }}_{f}\;f_{fu}\;\left(A_{fv}/s_{f}\right)\left(\cot\;\theta \;+\;\cot\;\beta \right)\;\sin\;\beta ]\;+\;[r\;{\tilde{\sigma }}_{s}\;f_{yt}\;\left(A_{v}/s\right)\left(\cot\;\theta \;+\;\cot\;\alpha \right)\;\sin\;\alpha \right]\right\}$$

(10) $$V=\left(b_{w}\;0.9\;d\;0.5\;f_{c}\right)\;\left\{\left[{\tilde{\sigma }}_{c}\;\left(\cot\;\theta +\cot\;\alpha \right)\;\sin^{2}\;\theta ]+[R\;{\tilde{\sigma }}_{f}\;f_{fu}\;\left(A_{fv}/s_{f}\right)\left(\cot\;\beta -\cot\;\alpha \right)\;\sin\;\beta \right]\right\}$$

(11) $$V=\left(b_{w}\;0.9\;d\;0.5\;f_{c}\right)\;\left\{\left[{\tilde{\sigma }}_{c}\;\left(\cot\;\theta +\cot\;\beta \right)\;\sin^{2}\;\theta ]+[r\;{\tilde{\sigma }}_{s}\;f_{yt}\;\left(A_{v}/s\right)\left(\cot\;\alpha -\cot\;\beta \right)\;\sin^{2}\;\alpha \right]\right\}$$

In the equations above, $\tilde{\sigma }$*_f_ = σ_f_/f_fu,_* and $\tilde{\sigma }$*_s_ = σ_s_/f_yt_* are the non-dimensional stresses of the FRP reinforcement and steel stirrups, respectively. *R* is the coefficient for effective strain and stress for the FRP at failure, where the effective stress is *f_fe_* = *f_fu_ R* = *E_f_ ɛ_fe_* and the effective strain is *ɛ_fe_* = *ɛ_fu_* *R*; *f_fu_* is the ultimate stress of the fiber; *r* is the efficiency coefficient for the steel stirrups, which considers the efficiency of the steel stirrups involved by the shear-critical crack; *β* represents the angle of the FRP and *α* represents the angle of the shear reinforcement with the beam axis.

The static theorem of plasticity is used to evaluate the shear strength of RC beam. It means that the shear strength is the maximum value among solutions, and it should satisfy all the equations including the plastic admissibility equations given below:

(12) $$0\;\leq \;{\tilde{\sigma }}_{c},\;{\tilde{\sigma }}_{f}\;\leq \;1,\;-1\;\leq \;{\tilde{\sigma }}_{c}\;\leq \;1$$

By combining (9), (10) and (12), Equation (13) is obtained. It shows the relation between the FRP, the transverse steel reinforcement and the stress field of the concrete strut.

(13) $$0\;\leq \;{\tilde{\sigma }}_{c}=\;\left(R{\tilde{\sigma }}_{f}\;A_{fu}\;f_{fu}/\left(b_{w}\;s_{f}\;0.5\;f_{c}\right)\;\sin\;\beta +r\;{\tilde{\sigma }}_{s}\;A_{v}\;f_{yt}/\left(b_{w}\;s\;0.5\;f_{c}\right)\;\sin\;\alpha \right)\;\left(1+\cot^{2}\;\theta \right)\;\leq \;1$$

According to the code’s suggestion, the lower limit of cot *θ* = 1.0, and the upper limit of cot *θ* = 2.5 hold. Based on this limitation of inclination of the concrete strut, the shear strength can be evaluated in two steps: (1) Initially, it is assumed that at the failure phase all the three stress fields could reach their stress limit simultaneously. Hence, using the inequality given in Equation (13), the inclination of the concrete strut can be evaluated as:

cot *θ =* ((*R A_fv_ f_fu_/*(*b_w_ s_f_* 0.5 *f_c_*) sin *β + r A_v_ f_yt_/*(*b_w_ s 0.5 f_c_*) sin *α*)^−1^ – 1)^1/2^ (14)

Step 2: three different cases can occur, depending on the amount of steel or FRP shear reinforcement mechanical ratio *ω_sw_* and *ω_fw_*, respectively.

Case 1 (small *ω_sw_* and *ω_fw_* values):

cot *θ* > 2.5: It implies that cot *θ* = 2.5 must be assumed, and the concrete strut does not fail due to the presence of a small amount of shear reinforcement. So, the shear strength is calculated using Equation (9), in which $\tilde{\sigma }$*_f_* = $\tilde{\sigma }$*_s_* =1, while the stresses on the concrete strut can be obtained by using Equation (13). 

Case 2 (intermediate *ω_sw_* and *ω_fw_* values):

1 ≤ cot *θ* ≤ 2.5: It implies that all the stress fields simultaneously achieve their stress limit. Equation (9) is thus used to find the shear strength considering $\tilde{\sigma }$*_f_* = $\tilde{\sigma }$*_s_ =* $\tilde{\sigma }$*_c_ =* 1. 

Case 3 (very large *ω_sw_* and *ω_fw_* values):

cot *θ* < 1: It implies that cot *θ* = 1.0 must be assumed, and the failure is due to reaching the stress limit in the concrete strut and in one of the shear reinforcements. If it is assumed that *β < α*, then the maximum shear strength of the beam is given by the minimum value obtained via Equation (10) assuming that the FRP reinforcement attains the maximum effective strain in tension $\tilde{\sigma }$*_f_ =* 1 or by Equation (11), considering that the yielding in the steel stirrups is attained, having $\tilde{\sigma }$*_s_* = 1. In Appendix B, the above three different cases are elucidated by calculation examples.

## 3. Reduction Factors for Steel Stirrups “*r*”

Different research has revealed that the simultaneous presence of FRP and steel stirrups decreases the contribution to the shear strength provided by shear reinforcement. It was also found that the increase in the axial rigidity ratio between the steel and FRP causes a reduction in the shear contribution provided by the externally bonded FRP [12,13,31,32,33].

Due to this interaction, some models were developed; the one proposed by Modifi and Chaallal [34] considers the interaction between the two reinforcement systems and their rigidities, while the model developed by Pellegrino and Modena [32] assumes a fixed reduction coefficient.

In order to model the interaction between the FRP and steel stirrup, Colajanni et al. [19] also included a similar factor in their model, which is able to take into account the possible different inclinations of the FRP and the pre-existing steel web reinforcement. “*r*” is defined as a bilinear expression that links the reduction in the contribution to the shear strength provided by transverse reinforcement to the ratio between the FRP effective strain in the direction of the steel reinforcement *ɛ_fe,s_ = ɛ_fe_* cos(*α − β*) and the yield strain of the steel stirrup (*ɛ_syw_*). If *ɛ_fe,s_*/*ɛ_syw_* ≤ 1.33, then *r =* 0.75 *ɛ_fe,s_*/*ɛ_syw_*, otherwise it is considered as *r =* 1.

## 4. Effectiveness Factor “*R*”

The failure of a shear-strengthened RC beam with externally bonded FRP is due to several factors, including peeling of the concrete cover, failure of the FRP, debonding of the FRP from the concrete surface, the loss of aggregate interlock, etc. Most of these phenomena precede attainment of the ultimate strain in the FRP. Thus, in order to limit the contribution of the FRP reinforcement, the effectiveness factor “*R*” is applied to the ultimate strain of the FRP fiber, which reduces the ultimate FRP tensile stresses.

To evaluate shear strength, six different effectiveness factors “*R*” were used. All of these have different approaches to deal with the strengthening of FRP. The first two *R* factors were used in [19]; the first was derived according to Khalifa and Nanni and Pellegrino and Modena [22,23,24]. The effectiveness factor is taken as the minimum among the four coefficients (*R*_1_, *R*_2_, *R*_3_, *R*_4_), which represent different modes of failure. *R*_1_ considers the tensile failure of the FRP, while *R*_2_ and *R*_3_ represent the debonding phenomenon and failure of the FRP due to shear crack width, respectively. Lastly, *R*_4_ considers failure due to peeling of the concrete cover.

*R*_1_ = 0.56(*ρ_f_ E_f_*)^2^ − 1.22(*ρ_f_ E_f_*) + 0.78

*R*_2_ = [(*f_ck_*)^2/3^(*d_fv_* − *ηL_e_*) [738.93 − 4.06(*E_f_ t_f_*)]]/*ε_fu_d_fv_*10^6^

*R*_3_ = 6 × 10^−3^/*ε_fu_*

*R*_4_ = (2*f_ct_A_c_*cos^2^*βb_c,v_*)/(*n_f_ t_f_ L_f_ E_f_* [(*h_f_* − L_e_)/(*h_f_*)]b_f_ ε_fu_)

The second *R* factor was proposed by Chen and Teng [7]. It is the minimum between two factors (*R*_5_ and *R*_6_). One represents the tensile rupture of the FRP across the crack, and the other represents the debonding failure of the FRP due to insufficient bond length.

*R*_5_ = (1 + (*d*
*− d_fv_*)/z)/2

For *λ* < 1 (*λ* = *L_max_*/*L_e_*)

*R*_6_ = (σ*_f,max_*/*E_f_*
*ε_fu_*) × (2/*π* λ × ((1−cos *π λ*/2)/(sin *π λ* /2)))

For λ ≥ 1

*R*_6_ = (*σ_f,max_*_/_*E_f_*
*ε_fu_*) × (1 − (*π*−2)/*π λ*)

The definition of the other four effectiveness factors can be found in ACI [18], CNR [21] fib [35] and Mofidi and Challal (M&C) [36].

## 5. Description of Data Sets and Analysis Steps

**Figure 3:**
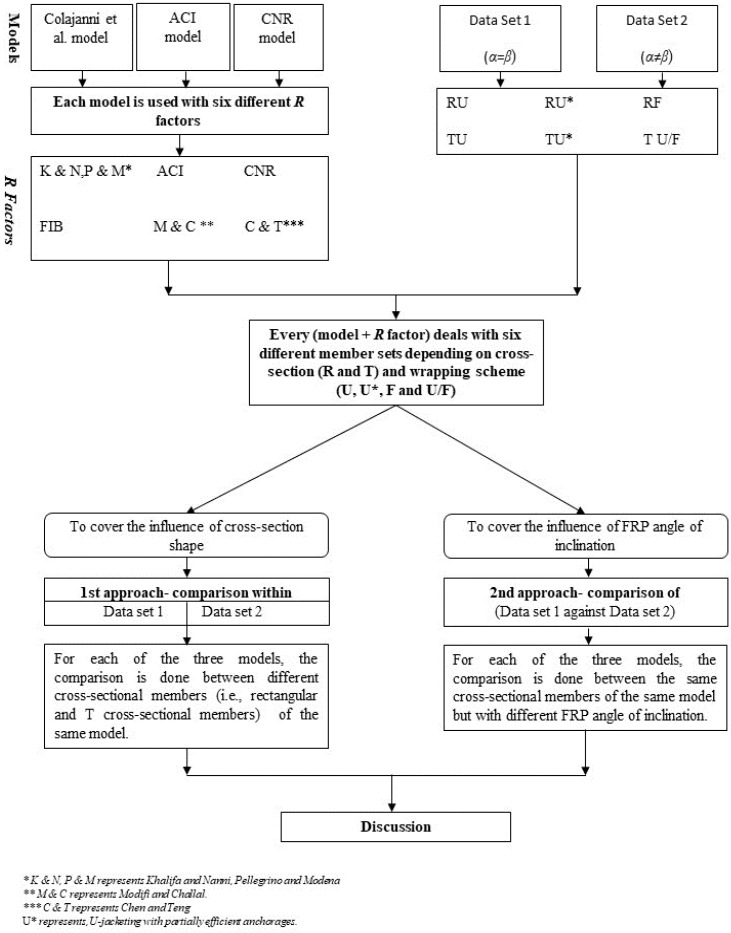
Flow chart representing data-analysis methodology.

To cover the main aspects of the research (influence of cross-section shape and angle of FRP inclination), two data sets and two comparison approaches were adopted which are explained in Figure 3. The beams included in the database have effective depths of the cross section in the range of 155 mm and 831 mm, while the shear span ranges between 2.3 m and 3.8 m. The transverse internal steel reinforcement is constituted by vertical steel stirrups whose maximum geometrical ratio is 0.48%. 

The FRP reinforcement geometrical ratio varies between 0.04% and 3.00%. The ultimate FRP tensile strength ranges between 106 and 4361 MPa, while the Young’s modulus varies between 8 and 640 GPa. In order to stress the influence of the FRP inclination (*β*), two data sets were analyzed, namely Data Set 1 (DS1) with *α* = *β* and Data Set 2 (DS2) with *α* ≠ *β*, where *α* is the inclination of the steel reinforcement with the beam axis while *β* represents the angle of the FRP with the beam axis. 

Both data sets contain results regarding R and T members with different wrapping schemes. On the basis of the wrapping scheme, rectangular RC beams are divided into three subsets, namely RF, RU and RU*. The first subset F represents full/complete wrapping, the U represents U-jacketing without anchorages, and U* represents U-jacketing with partially efficient anchorages. Analogously, T beams are divided into TU, TU* and TU/F, the latter representing U-jacketing with fully efficient anchorages. 

It is pointed out that, for the U*-wrapping scheme, the shear strength was assessed as if the beam was strengthened by ordinary U-jacketing, since the increase in shear capacity provided by partially efficient anchors cannot be assessed. Moreover, in the case of U/F, the beams were considered as strengthened by complete wrapping (as done in [20]).

In (DS1), where *α* = *β*, there are 40 rectangular beams with U-shaped wrapping, 7 reinforced-concrete beams with partially efficient anchorages (U*), and 10 beams with the complete/full wrapping scheme. Similarly, there are 52 T reinforced beams with U-wrapping, 18 T beams with U*-wrapping, and 11 TU/F beams with fully efficient anchorage.

In (DS2), where *α ≠ β,* there are 10 rectangular beams with U-wrapping, 7 rectangular reinforced-concrete beams RU* with partially efficient anchorages and 1 rectangular beam with complete wrapping. In DS2, for T beams there are two beams with U-wrapping, while for TU* and T U/F no experimental shear values are available. 

During the analysis, *v_exp_* of the data sets was used. *v_the_* is the analytical assessment of dimensionless shear, which for each model was calculated using all six different *R*-factor models, while *v_exp_* is the experimental value which can be expressed as *v_exp_ = V_exp_*/(*b_w_* 0.9*d* 0.5 *f_c_*). *τ_avg_* is the average value of the ratio *τ_avg_ = V_exp_*/*V_the_*.

The following steps are performed in the analysis:

1. Colajanni et al. Model [19], ACI model [18] and CNR model [21] are used for the analysis with six different *R* factors.
2. Every model + *R* factor deals with six different member sets, which differ in the type of cross section (R and T) and wrapping scheme (U, U*, F and U/F).
3. For each data set, the results provided by the Colajanni et al. model with the six different formulations of the *R* effectiveness factor are discussed.
4. For each of the three models: in the first approach, to cover the influence of the cross-section shape, a comparison is made between the R and T sections (i.e., between RU and TU, between RU* and TU*, between RF and TU/F within Data Set 1 and within Data Set 2).
5. For each of the three models: in the second approach, to recognize the influence of the FRP inclination angle, a comparison is made of Data Set 1 against Data Set 2 for the effectiveness of each model in the strength assessment of members having the same cross-section shape but with different inclination angles (i.e., RU of DS1 and RU of DS2, RU*of DS1 and RU* of DS2, RF of DS1 and RF of DS2).

## 6. Results and Discussion 

Different effectiveness factors (*R*) are used in the model proposed by Colajanni et al. [19] and their efficiency is compared in order to determine the influence of the effectiveness factor on the shear capacity assessment. To evaluate the reliability and efficiency of the model, the average ratio *τ_Avg_* = *V_exp_*/*V_the_* and its CoV are analyzed. In the reliability assessment, *R* as proposed by ACI [18], CNR [21], fib [35] and Mofidi and Challaal [36] (M&C) is used in its original form, considering the steel-stirrup reduction factor equal to r = 1, as well as two *R*-factor models proposed in Colajanni et al. [19] including the steel-stirrup reduction factor. 

In Table 1, the results for the whole database reported in Table A1 of Appendix A are summarized, proving that the *R* factor has a major effect on evaluating the shear strength of strengthened beams. By using the effectiveness factor proposed by CNR or ACI, the best average values (mean efficiency ratio *τ_avg_* = 0.97) were obtained, while the worst results were obtained in the case of using the *R* factor of fib. 

The M&C model yielded a 12% underestimation of the average shear strength. The effectiveness factors of Khalifa and Nanni + Pellegrino and Modena, and Chen and Teng yielded better results as compared to fib but with a slight overestimation of the shear strength. A coefficient of variation parameter was also used to analyze the results. From the results, it is seen that the highest scattering of data was observed in the case of *R* as given by the ACI model with (CoV = 0.32) while it is concluded that the highest accuracy and reliability was obtained in the case of the Chen and Teng *R*-factor model, since it provides the smallest CoV (0.20) with a *τ_avg_* = 0.95, very close to the best ones.

**Figure 4:**
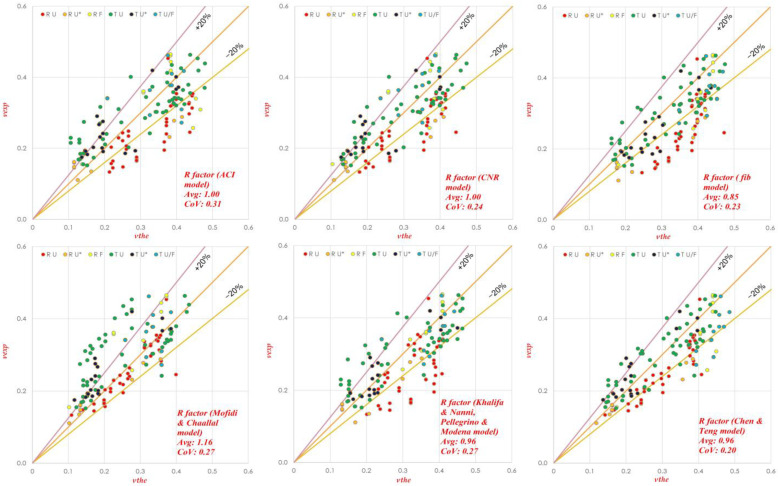
Experimental vs. theoretical shear strength for the Colajanni et al. model with six different *R* factors. (Data Set 1, *α* = *β*). RU* and TU* represents Rectangular and T beam having U-jacketing with partially efficient anchorages.

A more effective analysis can be performed if the whole database is split into two subsets, according to the values of the inclination of the steel (*α*) and FRP (*β*) web reinforcement. From Figure 4, it can be seen that, for *α* = *β*, similarly as for the whole database, a very accurate estimation of the shear strength was achieved by using the *R* factor of ACI and CNR, while the worst results were obtained in the case of using the effectiveness factor of fib and M&C. The *R*-factor model proposed by Chen and Teng slightly (4%) overestimated the shear strength, but less scattering was observed as (CoV = 0.20). 

**Figure 5:**
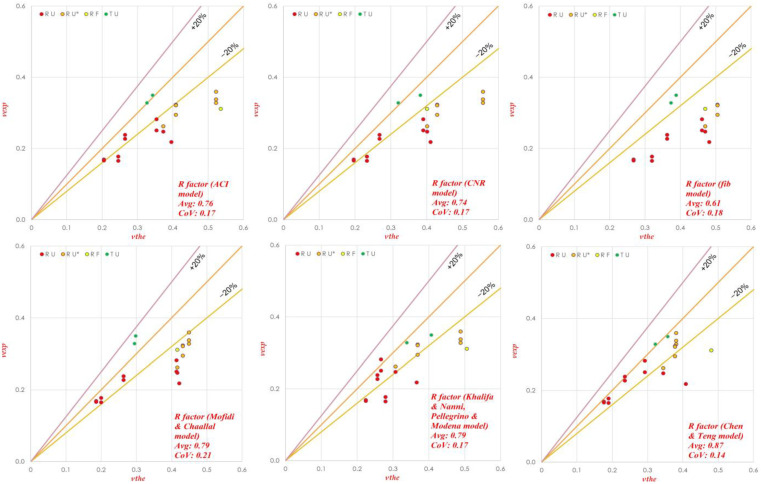
Shear strength calculation (experimental vs. theoretical) for the Colajanni et al. model with six different *R* factors. (For Data Set 2, *α ≠ β*). RU* represents Rectangular while TU* represents T beam having U-jacketing with partially efficient anchorages.

By contrast, from Figure 5, where *α* ≠ *β*, it can be seen that a large over estimation of shear strength was found for all the models; the largest one (*τ_avg_* = 0.61) as well as the largest scattering of data was observed when the *R* factor proposed by the fib model was used. Similar results can be observed in the case of *α* = *β*. 

The highest accuracy among the others with less overestimation and scattering (*τ_avg_* = 0.87, CoV = 0.14) was observed for the effectiveness factor proposed by Chen and Teng. This unfavorable large overestimation was less marked in the models with the effectiveness factors of Khalifa and Nanni + Pellegrino and Modena, or Chen and Teng because of the introduction of the “*r*” factor. In Table 2, the results are further subdivided into the two databases described in Section 6.1, Section 6.2 and Section 6.3.

### 6.1. Conclusion Based on 1st Approach for DS1 (α = β)

The results were compared by taking the average value provided by the six different *R*-factor models, divided on the basis of section shape and efficiency of the anchorage. In the Colajanni et al. model, the results in the case of RU revealed that there was an overestimation of the shear strength on average of about 19%, while in the TU scheme, the shear strength on average was underestimated by about 9%. The first lack of accuracy, namely the overestimation of the rectangular sections, can be attributed to the inefficiency of the model in taking into account the reduced length of the FRP fiber, which in many specimens did not reach the top end of the beam. For T beams, the overestimation was mitigated by the presence of the shear contribution provided by the flange; this contribution produced a significant increment in the strength of the tested specimens that was not predicted by the model.

In the case of RU* and TU*, there was the same trend as for RU and TU. There was almost a 13% overestimation on average in the case of RU* and 11% in the case of TU*. There was less of a dispersion of the data observed for TU* with an average CoV of 0.18.

In the case of fully wrapped rectangular or equivalent fully wrapped T sections, namely RF and TU/F, the average values were almost the same, and ***τ_avg_*** was close to unity. This is due to the fact that for RF and TU/F, some approaches underestimated while others overestimated the shear strength. It was observed that overall, more accurate estimation of the shear strength was obtained for RF and TU/F as compared to the previous comparisons, with less dispersion. This is due to the fact that the effective fiber strength of the completely wrapped sections was more accurately estimated than that of the partially wrapped ones, and possibly the partial ineffectiveness of the anchorage in the TU/F section was compensated by the flange contribution. 

Overall, the Colajanni et al. model slightly overestimated the shear strength for R-cross-sectional members while it underestimated it for T-cross-sectional members, but generally speaking, the underestimation in the case of the T members was less as compared to the overestimation of the R members. 

The ACI model predicted the results very accurately in the case of both RU- and TU-cross-sectional members. Just a 1% overestimation was observed in the case of the RU cross sections while a 3% overestimation was observed in the case of the TU members, which reveals that this model is less sensitive to the contribution of flanges. More accurate results were obtained by the C&T *R*-factor model with CoV = 0.14 for R members and CoV = 0.19 for T members. 

In the case of RU* there was less of a dispersion of the data (Avg value of the CoV = 0.08) while for TU* there was more of a dispersion of the data (CoV = 0.23). A 13% overestimation was observed in the case of RU* and a 6% underestimation for TU*.

In general, less accurate results were observed in the case of TU/F (Avg CoV= 0.33) due to the flange effect. Additionally, it was observed that the ACI model yielded better results in the case of the R member than the T-cross-sectional ones. When *α* = *β,* the CNR model yielded the same results as the Colajanni et al. model when the same value of *R* was used, and it also performed well enough in the prediction of the average shear strength of RF and TU/F.

### 6.2. Conclusion Based on First Approach for DS 2 (α ≠ β)

The Colajanni et al. model, similarly to the CNR model, in the case of RU overestimated the shear strength by about 25%, but it yielded a better estimation of the average shear strength in the case of TU. Actually, the results referring to the T database for (*α ≠ β*) were very few, with only two specimens for the TU series, so the values of CoV were almost meaningless. Additionally, due to the lack of data on TU*, a comparison cannot be made. 

In general, the ACI model was the performed best in the case of the rectangular beams, because it only overestimated the shear strength by about 18%, but it underestimated it by about 8% in the case of T members.

### 6.3. Conclusion Based on the 2nd Approach

A comparison of the effectiveness of each model in the strength assessment of specimens having the same cross-section shape but with different inclination angles stresses that both the Colajanni et al. and CNR models for RU and RU* overestimated more ***τ_avg_*** in DS2 than in DS1, but with less of a dispersion of the data, while the average overestimation for RU* was larger in the CNR model than in the Colajanni one. This is due to the ability of the Colajanni et al. model to take into account the difference of steel and FRP reinforcement orientation, and to properly evaluate their contributions in determining the inclination of the concrete strut. 

For TU, excellent results were obtained for both models in the case of DS2, with the best prediction of the average shear strength being only a 1% overestimation with less of a dispersion of the data (CoV 0.04) for both the Colajanni and CNR models. In the case of the ACI model, better results were obtained in the case of DS1 for RU and RU*, but in the case of TU, less accurate prediction but less of a dispersion was found in DS2. 

The CNR model provided the same results in the case of RU for both DS1 and DS2. In the case of RU* in DS1, it provided better values of ***τ_avg_***, but more scattering of the data as compared to DS2. In the case of TU, excellent results were achieved in DS2.

## 7. Conclusions

The large values of the CoV (up to 0.35) that affect the assessment of the shear strength in a large database that includes specimens with different cross-section shapes and FRP reinforcement inclinations suggest that a deep analysis of the results can provide insights into the merits and demerits of the analyzed models, as well as into the effect of different characteristics of the strengthened specimens. The analysis shows that most of the models are ineffective, with the exception of the ACI model (just 1% overestimation in the case of RU cross sections while a 3% overestimation in the case of TU), because of their inability to take into account a reduced height of the fiber with respect to the total effective depth of the section, due to the presence of the top flange of the section represented by the slab. Similarly, none of the analyzed models can adequately take into account the effect of the presence of flanges in the T section, which experimental results have proved to be effective in increasing the strength of the FRP-strengthened beam. 

Analyzing a database containing specimens with mechanical anchorages of the FRP, another source of uncertainty derives from the efficiency of the anchorages, which in many cases is not able to ensure that failure only occurs when the ultimate strain of the FRP is reached, i.e., by preventing FRP debonding failure.

Regarding the effect of FRP fiber inclination, the Colajanni et al. model is the only one among those based on the variable inclination of the action of the compressed concrete that is able to consistently take into account any different inclination of the FRP (*β*) and steel reinforcement (*α*). This characteristic makes it possible to mitigate the overestimation of the resistance (15% for R section and 1% for T section) that affects all the analysis models for *α* ≠ *β*. This circumstance is favored by the presence of the effectiveness factor of the steel reinforcement in the model, which takes into account the different orientation of the two reinforcements, and the consistent evaluation of the strength of the compressed concrete.

## Figures and Tables

**Table 1:** Evaluation of combined results of Data Set 1 and Data Set 2 using the Colajanni et al. model with different *R* factors.

	*R* (K&N, P&M)	*R* (ACI)	*R* (CNR)	*R* fib	*R* M&C	*R* C&T
*τ*_avg_	0.94	0.97	0.97	0.82	1.12	0.95
CoV	0.27	0.32	0.25	0.25	0.29	0.20

**Table 2:** Results of calculation on the basis of the 1st and 2nd approaches from Database 1 and Database 2.

For *α* = *β*	Colajanni et al. Model	***R*** **Factors**	**RU**		RU*		**RF**		For *α* = *β*	Colajanni et al. Model	***R*** **Factors**	**TU**		TU*		**TU/F**	
			***τ_avg_***	**CoV**	***τ_avg_***	**CoV**	***τ_avg_***	**CoV**				***τ_avg_***	**CoV**	***τ_avg_***	**CoV**	***τ_avg_***	**CoV**
		K&N, P&M ^1^	0.78	0.25	0.87	0.25	1.01	0.10			K&N, P&M	1.06	0.27	1.04	0.16	1.01	0.22
		ACI	0.79	0.24	0.92	0.33	1.01	0.24			ACI	1.10	0.31	1.16	0.22	1.09	0.29
		CNR	0.82	0.18	0.89	0.23	1.13	0.24			CNR	1.05	0.21	1.17	0.21	1.13	0.18
		FIB	0.70	0.21	0.71	0.17	0.89	0.14			FIB	0.97	0.20	0.91	0.15	0.85	0.13
		M&C ^2^	0.95	0.13	1.00	0.15	1.25	0.20			M&C	1.30	0.29	1.27	0.17	1.12	0.18
		C&T ^3^	0.82	0.13	0.84	0.15	0.88	0.17			C&T	1.04	0.19	1.10	0.15	0.81	0.11
		Average	0.81	0.19	0.87	0.21	1.03	0.18			Average	1.09	0.24	1.11	0.18	1.00	0.19
	ACI Model	K&N, P&M	0.99	0.21	0.90	0.11	1.10	0.08		ACI Model	K&N, P&M	0.95	0.19	1.03	0.20	0.91	0.30
		ACI	0.95	0.15	0.86	0.09	1.06	0.26			ACI	0.96	0.19	1.07	0.24	0.96	0.35
		CNR	0.99	0.15	0.86	0.08	1.16	0.17			CNR	0.96	0.18	1.07	0.23	1.00	0.32
		FIB	0.91	0.17	0.79	0.07	0.94	0.10			FIB	0.94	0.19	1.00	0.23	0.83	0.34
		M&C	1.10	0.18	0.95	0.07	1.26	0.11			M&C	1.04	0.20	1.11	0.23	1.02	0.38
		C&T	1.01	0.14	0.88	0.06	0.91	0.15			C&T	0.98	0.19	1.07	0.22	0.77	0.29
		Average	0.99	0.17	0.87	0.08	1.07	0.15			Average	0.97	0.19	1.06	0.23	0.91	0.33
	CNR Model	K&N, P&M	0.78	0.25	0.87	0.25	1.01	0.10		CNR Model	K&N, P&M	1.06	0.27	1.04	0.16	1.01	0.22
		ACI	0.79	0.24	0.92	0.33	1.01	0.24			ACI	1.10	0.31	1.16	0.22	1.09	0.29
		CNR	0.82	0.18	0.89	0.23	1.13	0.24			CNR	1.05	0.21	1.17	0.21	1.13	0.18
		FIB	0.70	0.21	0.71	0.17	0.89	0.14			FIB	0.97	0.20	0.91	0.15	0.85	0.13
		M&C	0.95	0.13	1.00	0.15	1.25	0.20			M&C	1.30	0.29	1.27	0.17	1.12	0.18
		C&T	0.82	0.13	0.84	0.15	0.88	0.17			C&T	1.04	0.19	1.10	0.15	0.81	0.11
		Average	0.81	0.19	0.87	0.21	1.03	0.18			Average	1.09	0.24	1.11	0.18	1.00	0.19
For *α* ≠ *β*	Colajanni et al. Model	K&N, P&M	0.79	0.20	0.79	0.11	0.62	xxxxx	For *α* ≠ *β*	Colajanni et al. Model	K&N, P&M	0.91	0.09	xxxxx	xxxxx	xxxxx	xxxxx
		ACI	0.75	0.14	0.71	0.09	0.58	xxxxx			ACI	1.01	0.01	xxxxx	xxxxx	xxxxx	xxxxx
		CNR	0.74	0.16	0.67	0.10	0.78	xxxxx			CNR	0.97	0.08	xxxxx	xxxxx	xxxxx	xxxxx
		FIB	0.57	0.11	0.58	0.07	0.66	xxxxx			FIB	0.89	0.02	xxxxx	xxxxx	xxxxx	xxxxx
		M&C	0.77	0.20	0.73	0.08	0.75	xxxxx			M&C	1.14	0.04	xxxxx	xxxxx	xxxxx	xxxxx
		C&T	0.88	0.17	0.85	0.07	0.65	xxxxx			C&T	1.00	0.03	xxxxx	xxxxx	xxxxx	xxxxx
		Average	0.75	0.16	0.72	0.09	0.67	xxxxx			Average	0.99	0.04	xxxxx	xxxxx	xxxxx	xxxxx
	ACI Model	K&N, P&M	0.84	0.12	0.90	0.06	0.60	xxxxx		ACI Model	K&N, P&M	1.04	0.01	xxxxx	xxxxx	xxxxx	xxxxx
		ACI	0.83	0.13	0.83	0.07	0.56	xxxxx			ACI	1.09	0.05	xxxxx	xxxxx	xxxxx	xxxxx
		CNR	0.82	0.14	0.79	0.08	0.78	xxxxx			CNR	1.07	0.02	xxxxx	xxxxx	xxxxx	xxxxx
		FIB	0.71	0.13	0.71	0.07	0.65	xxxxx			FIB	1.04	0.06	xxxxx	xxxxx	xxxxx	xxxxx
		M&C	0.82	0.16	0.85	0.08	0.75	xxxxx			M&C	1.13	0.06	xxxxx	xxxxx	xxxxx	xxxxx
		C&T	0.88	0.13	0.95	0.09	0.64	xxxxx			C&T	1.09	0.04	xxxxx	xxxxx	xxxxx	xxxxx
		Average	0.82	0.14	0.84	0.07	0.66	xxxxx			Average	1.08	0.04	xxxxx	xxxxx	xxxxx	xxxxx
	CNR Model	K&N, P&M	0.79	0.20	0.78	0.13	0.57	xxxxx		CNR Model	K&N, P&M	0.89	0.12	xxxxx	xxxxx	xxxxx	xxxxx
		ACI	0.75	0.15	0.68	0.11	0.54	xxxxx			ACI	1.01	0.01	xxxxx	xxxxx	xxxxx	xxxxx
		CNR	0.74	0.17	0.63	0.10	0.76	xxxxx			CNR	0.97	0.08	xxxxx	xxxxx	xxxxx	xxxxx
		FIB	0.56	0.13	0.54	0.07	0.61	xxxxx			FIB	0.89	0.01	xxxxx	xxxxx	xxxxx	xxxxx
		M&C	0.76	0.21	0.68	0.06	0.72	xxxxx			M&C	1.14	0.04	xxxxx	xxxxx	xxxxx	xxxxx
		C&T	0.87	0.17	0.85	0.07	0.61	xxxxx			C&T	1.00	0.03	xxxxx	xxxxx	xxxxx	xxxxx
		Average	0.75	0.17	0.69	0.09	0.64	xxxxx			Average	0.98	0.05	xxxxx	xxxxx	xxxxx	xxxxx

K&N, P&M ^1^ represents Khalifa and Nanni, Pellegrino and Modena, MC ^2^ represents Modifi and Challal. C&T ^3^ represents Chen and Teng. U* represents beam having U-jacketing with partially efficient anchorages.

## Data Availability

The data presented in this study are available on request from the corresponding author.

## Abbreviations

Following Notations are used in this paper:

*A_fv_*, *A_sw_*	Area of steel stirrups
*R*	Reduction coefficient (ratio of effective average stress/strain in FRP sheet to its ultimate strength)
R	Rectangular beam
T	T beam
*V_c_*	Shear resistance of concrete
*V_s_*	Shear resistance of steel stirrups
*V_f_*	Shear resistance provided by FRP
*V_Rd,f_*	FRP contribution to the shear capacity
*V_Rd,s_*	Steel contribution to the shear capacity
*V_Rd,c_*	Steel contribution to the shear capacity
*τ_avg_*	Average shear strength (τ_avg_ = V_exp_/V_the_)
*α*, *β*	Angle of steel and FRP transverse reinforcement
*a*	Shear span
*b_f_*, *b_w_*	Web widths of FRP and concrete
*d*, *d_fv_*	Effective depth of beam and FRP
*f_c_*, *f*′*_c_*	Characteristic compressive strength of concrete
*E_f_*, *E_sw_*	FRP and steel elastic modulus
*f_bd_*	Design resistance of the adhesion between FRP and concrete
*f_ywd_*	Design steel stirrup strength
*f_fed_*	Effective design strength of the FRP shear reinforcement
*f_yt_*	Characteristic yield strength of transverse reinforcement
*f_fe_*	Effective stress in the FRP; stress level attained at section failure
*f_fu_*	Design ultimate tensile strength of FRP
*f_ck_*, *f_ctm_*	Characteristic cylinder compressive and mean concrete tensile strength of concrete
*f_sy_*, *f_yt_*	Yielding stresses of longitudinal steel reinforcement and steel stirrups
*h_w_*	Beam cross-section height
*k_v_*, *k*_1_, *k*_2_	Bond-reduction coefficient and modification factors
*L_max_*, *L_e_*	Maximum and effective length
*r*	Reduction factor for steel stirrups
*w_f_*	Spacing, thickness, and width of the FRP strip
*s_f_*, *t_f_*	Spacing and thickness of FRP strip
$\bar{s}$_f_	Spacing of FRP strips measured perpendicular to FRP strip axis
*s*	Spacing of the steel stirrups
*V*, *V_n_*	External, and nominal shear forces
*v_exp_*, *v_the_*	Experimental and theoretical nondimensional shear strengths, where *v_exp_* = (*V_exp_*/(*b_w_* 0.9*d* 0.5 *f_c_*))
*z*	Inner lever arm
*ɛ_syw_*	Yield strain of steel stirrup
*ɛ_fe_*	Effective FRP strain
*ɛ_fu_*	Nominal FRP strain
*ɛ_fe,s_*	Effective strain in the direction of transverse steel reinforcement
*θ*	Angle between member axis and concrete stress
*λ*	Maximum bond length (normalized)
$\tilde{\sigma }$_c_	Stress of the web concrete (non-dimensional)
$\tilde{\sigma }$_f_	Tensile stress of transverse FRP (non-dimensional)
$\tilde{\sigma }$*_s_*	Stress in transverse reinforcement (non-dimensional)
*φ*	Angle between the FRP reinforcement direction and steel stirrups
*ρ_f_*, *ρ_s_*	Transverse geometrical ratio of fiber and steel reinforcement
*σ_f,max_*	Maximum stress along the bond length
*ψ*	Fictitious angle of reinforcement incorporating FRP and transverse steel reinforcement
*ψ_f_* *ω_fw,_ ω_sw_* *ω_fw_* *ω_sw_*	Reduction factor equal to 0.95 in case of wrapping scheme, 0.85 for the other schemes Mechanical ratio of transverse FRP and stirrups reinforcement (2*b_f_t_f_f_fu_*)/(*b_w_* *s_f_* sin *β* *f_c_*) (*A_v_* *f_yt_*)/(*b_w_* *s* sin *α* *f_c_*)

## Appendix A. Specimen Details and Experimental Results

**Table A1:** Details of specimens along with the results obtained after experimental tests.

		*f_c_*	*b_w_*	*d*		*ρ_s_*	*f_yt_*	*E_sw_*	*t_f_*	*β*	*ρ_f_*	*f_fu_*	*E_f_*	*wrap*	*v_exp_*
	Specimen no.	(MPa)	(mm)	(mm)	a/d	(%)	(MPa)	(GPa)	(mm)	(°)	(%)	(MPa)	(GPa)	U,C	(-)
Sato et al. (1997) [37]	No.2	35.7	150	240	2.5	0.42	387	183	0.11	90	0.15	3480	230	T, U	0.39
	No.3	35.3	150	240	2.5	0.42	387	183	0.11	90	0.15	3480	230	T, U/C	0.46
Deniaud & Cheng (2001) [38]	T6S4-C90	44.1	140	528	2.8	0.10	520	260	0.11	90	0.08	3400	230	T, U	0.19
	T6S4-G90	44.1	140	528	2.8	0.10	520	260	1.80	90	2.57	106	18	T, U	0.20
	T6S2-C90	44.1	140	528	2.8	0.20	520	260	0.11	90	0.08	3400	230	T, U	0.21
Deniaud & Cheng (2003) [39]	T4S4-G90	30.0	140	362	3.0	0.10	520	200	1.80	90	2.57	106	18	T, U	0.30
	T4S2-G90	30.3	140	362	3.0	0.20	520	200	1.80	90	2.57	106	18	T, U	0.33
	T4S2-C45	29.4	140	362	3.0	0.20	520	200	0.70	45	0.50	442	45	T, U	0.33
	T4S2-Tri	30.4	140	362	3.0	0.20	520	200	2.10	60	3.00	124	8	T, U	0.35
Bousselham & Chaallal (2006) [40]	SB-S1-0.5L	25.0	152	356	3.0	0.38	650	215	0.06	90	0.08	3100	243	T,U	0.46
	SB-S1-1L	25.0	152	356	3.0	0.38	650	215	0.11	90	0.14	3100	243	T, U	0.42
	SB-S1-2L	25.0	152	356	3.0	0.38	650	215	0.21	90	0.28	3100	243	T, U	0.44
Pellegrino & Modena (2006) [24]	A-U1-C-17	41.4	150	250	3.0	0.39	534	210	0.17	90	0.22	3450	230	R, U	0.34
	A-U1-C-20	41.4	150	250	3.0	0.34	534	210	0.17	90	0.22	3450	230	R, U	0.32
	A-U1-S-17	41.4	150	250	3.0	0.39	534	210	0.17	90	0.22	3450	230	R, U	0.35
	A-U1-S-20	41.4	150	250	3.0	0.34	534	210	0.17	90	0.22	3450	230	R, U	0.34
	A-U2-C-17	41.4	150	250	3.0	0.39	534	210	0.33	90	0.44	3450	230	R, U	0.35
	A-U2-C-20	41.4	150	250	3.0	0.34	534	210	0.33	90	0.44	3450	230	R, U	0.33
	A-U2-S-17	41.4	150	250	3.0	0.39	534	210	0.33	90	0.44	3450	230	R, U	0.31
	A-U2-S-20	41.4	150	250	3.0	0.34	534	210	0.33	90	0.44	3450	230	R, U	0.30
Leung et al. (2007) [41]	SB-U1	27.4	75	155	2.9	0.28	550	210	0.11	90	0.10	4200	235	R, U	0.45
	SB-F1	27.4	75	155	2.9	0.28	550	210	0.11	90	0.10	4200	235	R, C	0.46
	SB-F2	27.4	75	155	2.9	0.28	550	210	0.11	90	0.10	4200	235	R, C	0.46
	MB-U1	27.4	150	305	3.0	0.28	550	210	0.22	90	0.10	4200	235	R, U	0.27
	MB-U2	27.4	150	305	3.0	0.28	550	210	0.22	90	0.10	4200	235	R, U	0.28
	MB-F1	27.4	150	305	3.0	0.28	550	210	0.22	90	0.10	4200	235	R, C	0.42
	MB-F2	27.4	150	305	3.0	0.28	550	210	0.22	90	0.10	4200	235	R, C	0.44
	LB-U1	27.4	300	660	2.7	0.14	550	210	0.44	90	0.10	4200	235	R, U	0.23
	LB-U2	27.4	300	660	2.7	0.14	550	210	0.44	90	0.10	4200	235	R, U	0.23
	LB-F1	27.4	300	660	2.7	0.14	550	210	0.44	90	0.10	4200	235	R, C	0.36
	LB-F2	27.4	300	660	2.7	0.14	550	210	0.44	90	0.10	4200	235	R, C	0.36
Monti & Liotta (2007) [12]	UF90	11.0	250	410	3.5	0.10	500	210	0.22	90	0.18	2600	390	R, U	0.25
	US60	11.0	250	410	3.5	0.10	500	210	0.22	60	0.08	2600	390	R, U	0.22
	US45+	11.0	250	410	3.5	0.10	500	210	0.22	45	0.09	2600	390	R, U	0.25
	US45++	11.0	250	410	3.5	0.10	500	210	0.22	45	0.06	2600	390	R, U*	0.26
	UF45+ A	11.0	250	410	3.5	0.10	500	210	0.22	45	0.12	2600	390	R, U*	0.33
	UF45++ B	11.0	250	410	3.5	0.10	500	210	0.22	45	0.12	2600	390	R, U*	0.34
	UF45++ C	11.0	250	410	3.5	0.10	500	210	0.22	45	0.12	2600	390	R, U*	0.36
	US45+ D	11.0	250	410	3.5	0.10	500	210	0.22	45	0.09	2600	390	R, U*	0.32
	US45++ E	11.0	250	410	3.5	0.10	500	210	0.22	45	0.09	2600	390	R, U*	0.32
	US45++ F	11.0	250	410	3.5	0.10	500	210	0.22	45	0.09	2600	390	R, U*	0.29
	WS45+	11.0	250	410	3.5	0.10	500	210	0.22	45	0.06	2600	390	R, C	0.31
	USVA	10.6	250	400	3.5	0.10	500	200	0.22	45	0.09	3000	390	R, U	0.25
	USVA+	10.6	250	400	3.5	0.10	500	200	0.22	45	0.09	3000	390	R, U	0.28
Pellegrino & Modena (2008) [32]	B-U1-C-14	46.2	150	240	3.0	0.48	534	210	0.17	90	0.22	3450	230	R,U	0.34
	B-U2-C-14	46.2	150	240	3.0	0.48	534	210	0.33	90	0.44	3450	230	R, U	0.35
	B-U1-C-17	46.2	150	240	3.0	0.39	534	210	0.17	90	0.22	3450	230	R, U	0.32
	B-U2-C-17	46.2	150	240	3.0	0.39	534	210	0.33	90	0.44	3450	230	R, U	0.33
Grande et al. (2009) [33]	RS4Wa	21.0	250	411	3.4	0.10	476	210	0.19	90	0.15	2600	392	R, C	0.26
	RS3Wa	21.0	250	411	3.4	0.13	476	210	0.19	90	0.15	2600	392	R, C	0.34
	RS2Wa	21.0	250	411	3.4	0.20	476	210	0.19	90	0.15	2600	392	R, C	0.31
	RS4Ub	21.0	250	411	3.4	0.10	476	210	0.19	90	0.15	2600	392	R, U*	0.23
	RS3Ua	21.0	250	411	3.4	0.13	476	210	0.19	90	0.15	2600	392	R, U*	0.28
	RS2Ua	21.0	250	411	3.4	0.20	476	210	0.19	90	0.15	2600	392	R, U*	0.29
Belarbi et al. (2012) [16]	RC-8-S90-NA	20.7	457	831	3.3	0.15	276	200	0.22	90	0.06	3792	228	T, U	0.24
	RC-8-S90-DMA	23.8	457	831	3.3	0.15	276	200	0.22	90	0.06	3792	228	T, U*	0.23
	RC-12-S90-NA	28.9	457	831	3.3	0.10	276	200	0.22	90	0.06	3792	228	T, U	0.15
	RC-12-S90-DMA	30.5	457	831	3.3	0.10	276	200	0.22	90	0.06	3792	228	T, U*	0.18
	RC-12-S90-PC	19.2	457	831	3.3	0.10	276	200	0.22	90	0.06	3792	228	T, U*	0.29
	RC-12-S90-HS-PC	18.3	457	831	3.3	0.10	276	200	0.22	90	0.06	3792	228	T, U*	0.27
Panda et al.(2013) [42]	S300-1L-SZ-U-90	40.4	100	230	3.2	0.19	252	200	0.36	90	0.72	160	13	T, U	0.22
	S300-1L-SZ-UA-90	40.4	100	230	3.2	0.19	252	200	0.36	90	0.72	160	13	T, U	0.23
	S200-1L-SZ-U-90	42.1	100	230	3.2	0.28	252	200	0.36	90	0.72	160	13	T, U	0.22
	S200-1L-SZ-UA-90	42.1	100	230	3.2	0.28	252	200	0.36	90	0.72	160	13	T, U	0.23
Baggio et al. (2014) [43]	6-G-N	50.1	150	310	2.9	0.21	384	200	0.51	90	0.34	575	26	R, U	0.16
	7-PD-G-N	50.1	150	310	2.9	0.21	384	200	0.51	90	0.34	575	26	R, U	0.15
	8-PD-G-CA	50.1	150	310	2.9	0.21	384	200	0.51	90	0.34	575	26	R, U*	0.15
	9-PD-G-GA	50.1	150	310	2.9	0.21	384	200	0.51	90	0.34	575	26	R, U*	0.16
Colalillo & Sheikh (2014) [44]	S5-US	47.6	400	545	3.1	0.07	501	195	1.00	90	0.25	961	95	R, U*	0.11
	S5-UA	47.6	400	545	3.1	0.07	501	195	1.00	90	0.50	961	95	R, U	0.13
	S5-CS	47.6	400	545	3.1	0.07	501	195	1.00	90	0.25	961	95	R, C	0.16
	S2-US	47.5	400	545	3.1	0.14	501	195	1.00	90	0.25	961	95	R, U*	0.13
	S2-UA	47.5	400	545	3.1	0.14	501	195	1.00	90	0.50	961	95	R, U	0.15
Ozden et al. (2014) [45]	FBwoA-CFRP	12.4	120	339	3.8	0.14	249	200	0.13	90	0.05	4300	238	T, U	0.27
	FBwA-CFRP	12.4	120	339	3.8	0.14	249	200	0.13	90	0.05	4300	238	T, U/C	0.36
	PBwA-CFRP	12.4	120	339	3.8	0.14	249	200	0.13	90	0.05	4300	238	U, U/C	0.29
	FBwoA-GFRP	12.4	120	339	3.8	0.14	249	200	0.16	90	0.06	3400	73	T, U	0.27
	FBwA-GFRP	12.4	120	339	3.8	0.14	249	200	0.16	90	0.06	3400	73	T, U/C	0.34
	PBwA-GFRP	12.4	120	339	3.8	0.14	249	200	0.16	90	0.06	3400	73	T, U/C	0.34
	FBwoA-Hi-CFRP	12.4	120	339	3.8	0.14	249	200	0.14	90	0.05	2600	640	T, U	0.24
	FBwA-Hi-CFRP	12.4	120	339	3.8	0.14	249	200	0.14	90	0.05	2600	640	T, U/C	0.27
	PBw-Hi-C	12.4	120	339	3.8	0.14	249	200	0.14	90	0.05	2600	640	T, U/C	0.31
Mofidi & Chaallal (2014) [36]	WT-ST-50	31.0	152	350	3.0	0.38	540	206	0.11	90	0.07	3450	230	T, U	0.33
	WT-ST-70	31.0	152	350	3.0	0.38	540	206	0.11	90	0.10	3450	230	T, U	0.34
	WT-SH-100	31.0	152	350	3.0	0.38	540	206	0.11	90	0.14	3450	230	T, U	0.34
Mofidi et al. (2014) [46]	S1-LS-NE	33.7	152	350	3.0	0.38	650	205	2.00	90	0.60	1350	90	T, U	0.34
	S1-LS-PE	33.7	152	350	3.0	0.38	650	205	2.00	90	0.60	1350	90	T, U*	0.37
	S1-EB-NA	33.7	152	350	3.0	0.38	650	205	0.11	90	0.14	3450	230	T, U	0.36
El-Saikaly et al. (2015) [47]	S1-EB	28.0	152	350	3.0	0.25	580	200	0.38	90	0.50	894	65	T, U	0.32
	S1-LS	28.0	152	350	3.0	0.25	580	200	1.40	90	0.21	2250	120	T, U	0.30
	S1-LS-Rope	28.0	152	350	3.0	0.25	580	200	1.40	90	0.21	2250	120	T, U/C	0.38
	S3-EB	28.0	152	350	3.0	0.38	580	200	0.38	90	0.50	894	65	T, U	0.38
	S3-LS	28.0	152	350	3.0	0.38	580	200	1.40	90	0.21	2250	120	T, U	0.36
	S3-LS-Rope	28.0	152	350	3.0	0.38	580	200	1.40	90	0.21	2250	120	T, U/C	0.42
Qin et al. (2015) [48]	S00	29.6	125	295	3.1	0.29	542	210	1.00	90	1.60	986	96	T, U	0.37
Chen et al. (2016) [49]	S8-U	46.1	200	320	3.0	0.25	416	200	0.17	90	0.08	4361	226	T, U	0.23
	S8-UFA1	46.1	200	320	3.0	0.25	416	200	0.17	90	0.08	4361	226	T, U*	0.24
	S8-UFA2	46.1	200	320	3.0	0.25	416	200	0.17	90	0.08	4361	226	T, U*	0.28
Frederick et al. (2017) [50]	TB2	27.2	130	235	3.2	0.17	415	200	0.15	90	0.23	1400	119	T, U	0.37
	TB4	27.2	130	235	3.2	0.17	415	200	0.15	90	0.23	1400	119	T, U*	0.42
El-Saikaly et al. (2017) [51]	EBS-BL	28.0	152	350	3.0	0.25	580	200	0.38	90	0.50	894	65	T, U*	0.37
	EBS-ER	28.0	152	350	3.0	0.25	580	200	0.38	90	0.50	894	65	T, U*	0.40
	EBL-RF	28.0	152	350	3.0	0.25	580	200	2.00	90	0.30	1350	90	T, U/C	0.4
	EBS-NA	28	152	350	3.0	0.25	580	200	0.38	90	0.5	894	65	T, U	0.32
	EBL-NA	28	152	350	3.0	0.25	580	200	2.00	90	0.3	1350	90	T, U	0.30
	EBL-RW	28	152	350	3.0	0.25	580	200	2.00	90	0.3	1350	90	T, U/C	0.38
Nguyen-Minh et al. (2018) [52]	P-A1-2.3-C	30.6	120	406	2.3	0.16	342	205	1.00	90	0.83	986	96	T, U	0.41
	P-A1-2.3-G	30.6	120	406	2.3	0.16	342	205	1.30	90	1.08	575	26	T, U	0.40
	P-A1-2.3-G-Cont.	30.6	120	406	2.3	0.16	342	205	1.30	90	2.17	575	26	T, U	0.43
	P-A1-2.3-C-Cont.	30.6	120	406	2.3	0.16	342	205	1.00	90	1.67	986	96	T, U	0.45
	P-A2-2.3-C	30.6	120	406	2.3	0.16	342	205	2.00	90	1.67	986	96	T, U	0.43
	P-B1-2.3-C	44.4	120	406	2.3	0.16	342	205	1.00	90	0.83	986	96	T, U	0.32
	P-B1-2.3-G	44.4	120	406	2.3	0.16	342	205	1.30	90	1.08	575	26	T, U	0.32
	P-B1-2.3-G-Cont.	44.4	120	406	2.3	0.16	342	205	1.30	90	2.17	575	26	T, U	0.34
	P-B1-2.3-C-Cont.	44.4	120	406	2.3	0.16	342	205	1.00	90	1.67	986	96	T, U	0.36
	P-B2-2.3-C	44.4	120	406	2.3	0.16	342	205	2.00	90	1.67	986	96	T, U	0.34
	P-C1-2.3-C	58.7	120	406	2.3	0.16	342	205	1.00	90	0.83	986	96	T, U	0.29
	P-C1-2.3-G	58.7	120	406	2.3	0.16	342	205	1.30	90	1.08	575	26	T, U	0.27
	P-C1-2.3-G-Cont.	58.7	120	406	2.3	0.16	342	205	1.30	90	2.17	575	26	T, U	0.31
	P-C1-2.3-C-Cont.	58.7	120	406	2.3	0.16	342	205	1.00	90	1.67	986	96	T, U	0.32
	P-C2-2.3-C	58.7	120	406	2.3	0.16	342	205	2.00	90	1.67	986	96	T, U	0.30
Oller et al. (2019) [20]	M1-a	42.8	200	493	3.0	0.12	646	200	0.17	90	0.04	3400	230	T, U	0.15
	M1-b	42.8	200	493	3.0	0.12	646	200	0.17	90	0.04	3400	230	T, U	0.15
	M1A	39.0	200	493	3.0	0.12	646	200	0.17	90	0.04	3400	230	T, U*	0.16
	M1B	38.5	200	493	3.0	0.12	646	200	0.17	90	0.04	3400	230	T, U*	0.17
	M2A	39.0	200	493	3.0	0.12	646	200	0.17	90	0.07	3400	230	T, U*	0.21
	M2B	38.5	200	493	3.0	0.12	646	200	0.17	90	0.07	3400	230	T, U*	0.21
	H1-a	44.4	200	493	3.0	0.12	646	200	0.17	90	0.04	3400	230	T, U	0.15
	H2-a	44.4	200	493	3.0	0.12	646	200	0.17	90	0.07	3400	230	T, U	0.19
	H2-b	49.7	200	493	3.0	0.12	646	200	0.17	90	0.07	3400	230	T, U	0.17
	H2A	44.7	200	493	3.0	0.12	646	200	0.17	90	0.07	3400	230	T, U*	0.19
	H2B	49.6	200	493	3.0	0.12	646	200	0.17	90	0.07	3400	230	T, U*	0.17
	H3A	44.7	200	493	3.0	0.12	646	200	0.17	90	0.17	3400	230	T, U*	0.23
	H3B	49.6	200	493	3.0	0.12	646	200	0.17	90	0.17	3400	230	T, U*	0.21
Alzate et al. (2013) [53]	U90S5-a(L)	37.0	250	420	3.5	0.11	500	200	0.29	90	0.14	4000	240	R, U	0.16
	U90S5-a(S)	37.0	250	420	3.5	0.11	500	200	0.29	90	0.14	4000	240	R, U	0.14
	U90S5-b(L)	28.0	250	420	3.5	0.11	500	200	0.29	90	0.14	4000	240	R, U	0.21
	U90S5-b(S)	28.0	250	420	3.5	0.11	500	200	0.29	90	0.14	4000	240	R, U	0.20
	U90C5-a(L)	24.5	250	420	3.5	0.11	500	200	0.29	90	0.23	4000	240	R, U	0.22
	U90C5-a(S)	24.5	250	420	3.5	0.11	500	200	0.29	90	0.23	4000	240	R, U	0.20
	U90C5-b(L)	22.6	250	420	3.5	0.11	500	200	0.29	90	0.23	4000	240	R, U	0.26
	U90C5-b(S)	22.6	250	420	3.5	0.11	500	200	0.29	90	0.23	4000	240	R, U	0.24
	U90S3-a(L)	20.5	250	420	3.5	0.11	500	200	0.17	90	0.08	3800	240	R, U	0.25
	U90S3-a(S)	20.5	250	420	3.5	0.11	500	200	0.17	90	0.08	3800	240	R, U	0.23
	U90S3-b(L)	22.6	250	420	3.5	0.11	500	200	0.17	90	0.08	3800	240	R, U	0.22
	U90S3-b(S)	22.6	250	420	3.5	0.11	500	200	0.17	90	0.08	3800	240	R, U	0.24
	U90S3-c(L)	28.0	250	420	3.5	0.11	500	200	0.17	90	0.08	3800	240	R, U	0.20
	U90S3-c(S)	28.0	250	420	3.5	0.11	500	200	0.17	90	0.08	3800	240	R, U	0.16
	U90C3-a(L)	30.2	250	420	3.5	0.11	500	200	0.17	90	0.13	3800	240	R, U	0.17
	U90C3-a(S)	30.2	250	420	3.5	0.11	500	200	0.17	90	0.13	3800	240	R, U	0.18
	U90C3-b(L)	30.2	250	420	3.5	0.11	500	200	0.17	90	0.13	3800	240	R, U	0.16
	U90C3-b(S)	30.2	250	420	3.5	0.11	500	200	0.17	90	0.13	3800	240	R, U	0.17
	U45S5(L)	30.7	250	420	3.5	0.11	500	200	0.29	45	0.14	4000	240	R, U	0.17
	U45S5(S)	30.7	250	420	3.5	0.11	500	200	0.29	45	0.14	4000	240	R, U	0.18

In the table, U* represents beam having U-jacketing with partially efficient anchorages.

## Appendix B. Calculation Examples of the Colajanni et al. Model

With regard to the shear model proposed by Colajanni et al. [19], in this section three calculation examples will be carried out using a step-by-step procedure based on the equations reported in Section 2.3. For each of the three possible cases (namely cot *θ* > 2.5, 1 ≤ cot *θ* ≤ 2.5, cot *θ* < 1) a calculation example is given below. With the exception of the third case (which is quite difficult to find in a real application since it would require an amount of fiber and/or stirrups not compatible with engineering applications), these examples are developed starting from one of the specimens listed in Appendix A, using the *R* factor based on the equations given in [7].

Case 1:

One of the specimens tested by Alzate et al. [53] is used. Beam U90S5-a(L) has a rectangular cross section with dimensions equal to 250 × 420 mm, and a length of 4300 mm. It has stirrups with a diameter of 8 mm arranged at a spacing of 380 mm, while it is retrofitted with a U-shaped scheme made with CFRP strips having a thickness of 0.29 mm, a width of 300 mm and a spacing of 500 mm, arranged at right angles with respect to the beam axis. Using the data reported in Appendix A, first of all the *R* factor is calculated according to the equations reported in Section 4 (i.e., *R*_5_ and *R*_6_, equal to 0.65 and 0.18, respectively). Then, starting from the *R*-factor value, the r factor is computed based on the procedure described in Section 3, equal to 0.92. After that, the inclination of the concrete strut is calculated via Equation (14), which provides a value of cot *θ* > 2.5; thus, due to the limitations of cot *θ* values, cot *θ* = 2.5 is assumed. Consequently, assuming $\tilde{\sigma }$*_f_* = $\tilde{\sigma }$*_s_* = 1, the shear capacity can be calculated using Equation (9), which provides a value of 310 kN.

Case 2:

One of the specimens tested by Pellegrino and Modena [24] is used. Beam A-U1-C-17 has a rectangular cross section with dimensions equal to 150 × 300 mm, and a length of 4800 mm. It has stirrups with a diameter of 8 mm arranged at a spacing of 170 mm, while it is retrofitted with a U-shaped scheme made with continuous CFRP sheets having a thickness of 0.17 mm, arranged at right angles with respect to the beam axis. Using the data reported in Appendix A, first of all the *R* factor is calculated according to the equations reported in Section 4 (i.e., *R*_5_ and *R*_6_, equal to 0.50 and 0.23, respectively). Then, starting from the *R*-factor value, the *r* factor is computed based on the procedure described in Section 3, equal to 1. After that, the inclination of the concrete strut is calculated via Equation (14), which provides a value of cot *θ* = 2.09. Therefore, assuming $\tilde{\sigma }$*_c_* = $\tilde{\sigma }$*_f_* = $\tilde{\sigma }$*_s_* = 1, the shear capacity can be calculated using one of Equations (9)–(11), which provide a value of 272 kN.

Case 3:

As already stated before, none of the specimens analyzed in the table reported in Appendix A provides a cot *θ* value lower than 1. Therefore, to carry out the comparison, the specimen with the lowest cot *θ* is selected and then the spacing of FRP is properly modified to obtain a cot *θ* less than 1. To this aim, one of the specimens tested by El-Saikaly et al. [47] is used. Beam S3-LS-Rope has a T cross section with dimensions equal to 152 × 406 mm, with a flange width and thickness of 508 mm and 102 mm, respectively. It has stirrups with a diameter of 8 mm arranged at a spacing of 175 mm, while it is retrofitted with a U-shaped scheme made with CFRP strips having a thickness of 1.4 mm, a width of 20 mm and a spacing of 175 mm, arranged at right angles with respect to the beam axis. Anchorages made with carbon-fiber ropes are added to each strip to prevent debonding failure. In fact, the experimental test showed an FRP tensile failure, equivalent to a complete wrapping scheme. Using the data reported in Appendix A, first of all the *R* factor is calculated according to the equations reported in Section 4 (i.e., only *R*_5_ because the section is considered fully wrapped thanks to the presence of the anchorages, and it is equal to 0.66). Then, starting from the *R*-factor value, the *r* factor is computed based on the procedure described in Section 3. After that, the inclination of the concrete strut is calculated via Equation (14), which provides a value of cot *θ* = 1.28. Therefore, the spacing of the CFRP is reduced to 110 mm, with which, again using Equation (14), a cot *θ* = 0.97 is obtained. Thus, cot *θ* = 1 is assumed, so Equation (10) is used considering $\tilde{\sigma }$*_f_* = 1 and Equation (11) is used with $\tilde{\sigma }$*_s_* = −1. The shear strength of the beam is the minimum one obtained through the above two equations, and it is equal to 335 kN.
